# Neuromorphic Devices: Materials, Structures and Bionic Applications

**DOI:** 10.3390/nano15171299

**Published:** 2025-08-22

**Authors:** Liqiang Zhu, Qing Wan

**Affiliations:** 1 School of Physical Science and Technology, Ningbo University, Ningbo 315211, China; 2 Yongjiang Laboratory, Ningbo 315202, China

With the recent developments in machine learning, Artificial Intelligence (AI), and Internet of Things (IoTs) technology, it is necessary to process massive amounts of data in an energy-efficient way. However, traditional computing architectures heavily rely on sequential processing, which not only consumes substantial amounts of power but also restricts the real-time execution of complex tasks [1]. Brain-inspired neuromorphic devices have attracted increased attention for addressing the dilemma. Designing neuromorphic devices is becoming an important branch of AI and neuromorphic engineering that will inject new vitality into the development of artificial intelligence in the future. With the development of new materials technology and new conceptual devices, several kinds of neuromorphic devices have been proposed [2,3,4,5,6,7]. Except for basic synaptic responses, advanced neural cognitive behaviors have been mimicked. Additionally, inspired by the powerful perceptual functions of human multi-sensory learning activities, hardware-based artificial perceptual systems have also been proposed by adopting neuromorphic devices and bio-inspired sensors [8,9,10,11,12]. Such perceptual systems demonstrate multi-sensing functions and show great potential in human–machine interfacing, humanoid robots, and next-generation cognitive wearable devices. All these achievements indicate the great potential of neuromorphic devices in neuromorphic engineering.

This Special Issue brings twelve articles, including six research articles and six review articles, dedicated to advanced neuromorphic devices and neuromorphic systems. The content of the Special Issue includes the following: neuromorphic computing hardware based on ITO/ZnO/HfOx/W bilayer-structured memory devices [13], a superconducting adiabatic neural network that implements XOR and OR Boolean functions [14], flexible organic electrochemical transistors for energy-efficient neuromorphic computing [15], nanoscale titanium oxide memristive structures for neuromorphic applications [16], defect-tolerant memristor crossbar circuits for local learning neural networks [17], binary-weighted neural networks using FeRAM array for low-power AI computing [18], emerging opportunities for 2D materials in neuromorphic computing [19], resistive switching devices for neuromorphic computing [20], oxide ionic neuro-transistors for bio-inspired computing [21], optical bio-inspired synaptic devices [22], electrolyte gated transistors for brain inspired neuromorphic computing and perception applications [23], and memristor-based spiking neuromorphic systems toward brain-inspired perception and computing [24]. Our Special Issue provides good references for the ongoing research and promotes the developments of hardware-based neuromorphic devices in terms of materials, structures, and bionic applications. It will be important for neuromorphic electronics and will be of interest to general readers of Nanomaterials.
